# Digitally deconvolving the tumor microenvironment

**DOI:** 10.1186/s13059-016-1036-7

**Published:** 2016-08-22

**Authors:** Dvir Aran, Atul J. Butte

**Affiliations:** Institute for Computational Health Sciences, University of California, Mission Hall, 550 16th Street, 4th Floor, Box 0110, San Francisco, CA 94158-2549 USA

## Abstract

Understanding a tumor’s complex cellular heterogeneity will be crucial for the development of better treatment strategies. A new study suggests a novel method for the in silico dissociation of solid tumors and presents novel insights that have implications for immunotherapy in cancer.

Please see the related Research article: www.dx.doi.org/10.1186/s13059-016-1028-7.

## Cellular heterogeneity of solid tumors

Traditionally, anti-cancer therapies have exclusively targeted malignant cancer cells. More recently, tumors are increasingly seen not just as a mass of proliferating cells but as a complex milieu of factors that promote and inhibit growth, as well as nutrients, chemokines, and, very importantly, other non-cancerous cell types. The cancer immunotherapy field has seen significant progress in targeting tumor-infiltrating lymphocytes (TILs) during the past decade. TILs are among a variety of innate and adaptive immune cells that interact with the malignant cancer cells to form a dynamic environment, in which they act to both promote and inhibit tumor growth, invasion, and metastasis [1]. A better understanding of the cellular heterogeneity of the tumor and of the interactions between the tumor and its microenvironment is crucial for improving existing treatments, for the discovery of predictive biomarkers, and for the development of novel therapeutic strategies.

The Cancer Genome Atlas (TCGA) provides us with the opportunity to study multiple “omics” characterizations of thousands of tumors across tens of cancer types and to associate these characterizations with clinical information from the patients [2]. This profoundly important dataset, which is open and accessible, has given rise to a surge in new knowledge about cancer. The cancer immunology field has attempted to leverage the TCGA dataset in developing methods that can accurately infer the heterogeneity and the components of the tumor microenvironment. Unfortunately, the high cellular and mutational heterogeneity of tumors make this a difficult problem to tackle. A simpler undertaking is to consider all the non-malignant cells in the tumor as a whole or, conversely, to estimate tumor purity—the percentage of transformed cancer cells in the tumor sample. Tumor purity can be inferred successfully by utilizing different types of measurements that are available in TCGA, such as copy number, transcriptional and DNA methylation landscapes, and also the actual available image slides. In a recent study, we demonstrated how even such a crude measure has an immense effect on genomic reasoning [3]. Thus, it is clear that studies of genomic data require the ability to resolve the tumor’s cellular heterogeneity. Deeper evaluation of the composition of cells in a tumor requires more complex and sophisticated methods that are associated with greater uncertainty.

## Computational methods for resolving a tumor’s cellular composition

In the past decade, many computational methods have been applied in an attempt to gain better insight into the cellular heterogeneity of bulk tumors (reviewed in [4]). Generally, these methods attempt to associate “reference” gene sets that have been learned from purified immune cell types with transcriptomic profiles. Several such methods look for the enrichment of gene signatures that are associated with these individual cell types. The most challenging problem is inferring the complete cellular composition of admixed transcriptomes, and several deconvolution techniques have been applied in an attempt to tackle this problem. A recent approach for enumerating cell proportions named CIBERSORT provided estimations for 22 immune subsets [5] and has since been widely applied to an array of cancer types. Deconvolution methods rely on a reference signature gene expression matrix for the inferred cell types. To date, these methods have been limited to microarray studies and thus are not directly applicable to TCGA.

To address this issue, Liu and colleagues combined deconvolution strategies with estimations of tumor purity and presented TIMER: Tumor-IMmune Estimation Resource, a novel method for estimating the proportions of tumor–immune infiltrating subsets [6]. In this method, tumor purity estimations are used to find and filter genes that are associated with immune infiltration, and deconvolution is then applied with improved certainty. To apply TIMER on TCGA samples, Liu and colleagues applied a method to remove “batch effects” on the signature matrix to allow direct estimation of the abundance of certain cell types. Importantly, to gain better accuracy, the authors followed the “less is more” philosophy, choosing to focus on six major immune cell subsets that are strongly distinguishable rather than on more cell types. Liu and colleagues assert that by including more immune cell types into the regression, CIBERSORT inference suffers from a statistical co-linearity that results in biased estimations.

## Application to immunotherapy treatments

Immunotherapy treatments have already helped vast numbers of patients who have cancers, such as melanoma and renal cell carcinoma, for whom traditional therapies have failed. Furthermore, ongoing studies suggest that these therapies may benefit patients who have many additional types of cancer, including lung, brain, head and neck, and stomach cancers [7]. Nevertheless, we do not yet know why immunotherapy is effective in some patients but not in others. Thus, there is a pressing need to develop better tools to distinguish patients who respond to cancer immunotherapy from those who do not. Careful examination of the associations between TIMER estimates and the known immunotherapy targets allowed Liu and colleagues to make interesting observations and to suggest novel targets for cancer vaccines. The authors found correlations between CD8^+^ T-cell levels and known cancer/testis antigens such as *MAGEA3*, which have been put forward as effective cancer vaccine targets. On the basis of this observation, they further suggested a novel target, *SPAG5*, as a potential vaccine target for multiple cancers. Another observation relates to *CTLA-4* expression and CD8^+^ T-cell abundance. *CTLA-4* is known to be expressed exclusively by T cells. There is an association between both *CTLA-4* expression and elevated CD8^+^ T cells and better clinical response to anti-*CTLA-4* treatment, but this association is relatively weak. In this study, the authors reported that a group of melanoma and renal cancer patients who had low CD8^+^ T cells unexpectedly expressed high levels of *CTLA-4*. This result may explain the varied clinical response to checkpoint blockade therapies, newer treatments that have no underlying anti-tumor effect but instead remove inhibition on immune cells targeting the cancer. Another immune checkpoint blockade gene, *TIM3*, showed a pattern of expression that was inversely related to T-cell numbers, a finding that was further validated by staining renal tumors, which showed its expression in cancer cells. The TIMER estimates are provided as a web resource to allow cancer immunologists to further explore the cancer–immune cell interactions (http://cistrome.org/TIMER).

## The road ahead

Characterization of the cellular composition of solid tissues was traditionally performed using flow cytometry-based methods. These are potent tools for immunology research and for monitoring changes in immune-cell quantities, but they require tissue destruction, thereby affecting cellular state, integrity, and accuracy. Another disadvantage of single-cell methods is the need to perform the analysis on fresh tissues, which requires a supporting operational system and does not allow the association of findings with known clinical outcomes. Thus, the emerging use of single-cell RNA sequencing will not yet allow us to profile the cellular composition of solid tissues accurately. Hence, computational algorithms that are used to deconvolve bulk transcriptomic profiles will continue to offer a parallel and powerful approach that makes it possible to infer changes in cell quantities from data describing gene expression in complex tissues. In silico tissue dissection can be performed on frozen and fixed tumor specimens, does not rely on single-cell suspension, and, most importantly, can be applied immediately to thousands of publicly available tumor samples.

While computational methods provide many advantages, they should be scrutinized carefully. All methods to date are based on reference transcriptomic profiles of pure immune cell types, which are used to infer the behavior of these cell types in admixtures. It is unclear if this is a valid hypothesis. For example, while a particular gene may be specific to one cell type and therefore will be assigned as a reference gene, the number of mRNA copies of that gene may vary significantly depending on the state of the tissue. In tumors, another layer of uncertainty is added to the modeling. In a recent study, we have shown that the cancer cells themselves may express notable inflammatory response genes, thus producing a pattern of expression that resembles that resulting from the infiltration of macrophages [8].

It is clear that current methods cannot yet capture the full cellular heterogeneity in tumors. Newer measurements using molecular imaging could provide the research community with better measurements from in vivo samples, whereas newer ways of measuring circulating free DNA and RNA might allow estimations of what is going on inside cancer cells. We envision that methods that can integrate information from complementary datasets—including imaging methods, genetic, epigenetic, transcriptomic and proteomics profiles, and immune repertoire profiles both of the bulk tumor and from single-cell analyses—will lead to comprehensive portrayal of tumors. In turn, we expect that this will lead to better treatment strategies and ultimately to better prognosis for patients.

## Abbreviations

TCGA, The Cancer Genome Atlas; TIL, tumor-infiltrating lymphocyte; TIMER, Tumor-IMmune Estimation Resource
